# Study on the burden of digestive diseases among Chinese residents in the 21st century

**DOI:** 10.3389/fpubh.2023.1314122

**Published:** 2024-01-10

**Authors:** Shijun Gong, Yuyu Zhang, Yaqiong Wang, Xianhu Yang, Baolian Cheng, Ziyi Song, Xingrong Liu

**Affiliations:** ^1^School of Public Health, Lanzhou University, Lanzhou, Gansu, China; ^2^Guangzhou National Laboratory, Guangzhou, Guangdong, China

**Keywords:** digestive diseases, APC model, ASR, EAPC, DALY

## Abstract

**Background:**

The global burden of digestive diseases has increased in recent years. The study aims to comprehend the trend of incidence and death rates related to digestive diseases in China from 2000 to 2020.

**Methods:**

The study collected data on digestive diseases and their causes, such as incidence rates, death rates, Years of Life Lost, Years Lived with Disability, Disability-Adjusted Life Years and estimated annual percentage change from the 2019 Global Burden of Disease website and the Chinese Health and Wellness Statistical Yearbook spanning. And we employed the age-period-cohort model to analyze the influence of age, period, and birth cohort on the trend of death rates associated with digestive diseases.

**Results:**

In contrast to the global burden of digestive disease, China experienced increases in the age-standardized incidence for inflammatory bowel disease, gallbladder and biliary diseases, as well as appendicitis from 2000 to 2019. The corresponding estimated annual percentage change for these diseases were 2.06, 1.74, and 0.99. Females showed a significantly higher incidence of digestive diseases, while males experienced a higher death rate. Moreover, individuals in the age groups under 5 years and over 60 years exhibited higher death rates than those in other age groups.

**Conclusion:**

The findings underscore the ongoing importance of digestive diseases as a substantial public health issue in China. Reducing the disease burden of IBD in China necessitates healthcare systems to enhance their infrastructure and personnel readiness, ensuring an equitable, affordable, and accessible distribution of care for IBD patients. To reduce the occurrence and mortality rates of digestive diseases in China, the government should promote the adoption of early screening policies for individuals under the 5 year and those above the 60 year. These policies should be accompanied by customized preventive measures.

## 1 Introduction

Digestive diseases encompass a wide range of conditions, including both organic and functional disorders that affect the esophagus, stomach, intestine, liver, gallbladder, pancreas, and other associated organs. They represent a significant and impactful cause of morbidity and mortality on a global scale. Globally, digestive diseases have a high incidence, characterized by sudden onset and considerable distress. In 2019, digestive diseases accounted for 18.2% of the incidence, 14.2% of death rate, and 10.9% of Disability-Adjusted Life Years (DALY) across diseases (1).

Fang Wang et al. reported that digestive diseases accounted for an estimated 443.53 million incident cases, 2.56 million deaths, and 88.99 million DALY globally in 2019. These numbers increased by 74.44, 37.85, and 23.47%, respectively, from 1990. Additionally, the age-standardized death rates (ASDR) and age-standardized DALY rates (ASDALYs) decreased by 1.38 and 1.32% respectively, while age-standardized incidence rates (ASIR) showed a slight increase (2). Digestive diseases account for 32.04% of global non-communicable disease incidence and 6.08% of non-communicable disease-related deaths worldwide. In 2019, cirrhosis of the liver and other chronic liver diseases, upper gastrointestinal diseases, Paralytic ileus and intestinal obstruction, and Gallbladder and biliary disease have higher mortality and DALY than other digestive diseases (3). In the United States, digestive diseases have a significant impact, leading to millions of hospital visits and hundreds of thousands of deaths each year. The associated cost reaches billions of dollars, leading to a significant diversion of healthcare resources (4, 5). Digestive diseases are influenced by a variety of factors, including individual lifestyle habits, psychological factors, environmental conditions, nutritional status, infections, and genetic factors. Research has identified smoking, alcohol consumption, substance abuse, and elevated body mass index (BMI) as significant risk factors for digestive diseases (6).

Previous studies indicate a gradual decrease in the incidence of digestive diseases in China from 1990 to 2019. An estimated annual percentage change (EAPC) for age-standardized incidence rates, death rates, Years of Life Lost (YLL) rates, Years Lived with Disability (YLD) rates, and DALY rates of digestive diseases during this period were − 0.01 (−0.08 to 0.07), −3.38 (−3.53 to 3.24), −3.99 (−4.12 to 3.87), −0.89 (−0.97 to 0.80), and − 3.26 (−3.34 to 3.17) (3). Data sourced from the China Health and Wellness Statistical Yearbook for 2021 indicates that digestive diseases accounted for 10.75% of all ailments among patients admitted to public hospitals in China, ranking third after circulatory system diseases (17.33%) and respiratory system diseases (11.88%) (7). The study aims to evaluate the burden of digestive diseases in China and estimate the impact of age, period, and cohort effects on death rates related to digestive diseases. This research provides a crucial reference for the development of public health strategies to reduce the burden of digestive diseases.

## 2 Materials and methods

### 2.1 Overview

We used Descriptive Study method in this study. The burden of disease was described by analyzing data on digestive diseases in China. As per the Global Burden of Disease Study (8), the digestive diseases is categorized into 10 groups. These include cirrhosis and other chronic liver diseases (due to hepatitis B, hepatitis C, alcohol use, nonalcoholic fatty liver disease, and other causes), Upper digestive system diseases (such as Peptic ulcer disease, Gastritis and duodenitis, and Gastroesophageal reflux disease), Appendicitis, Paralytic ileus and intestinal obstruction, Inguinal, femoral, and abdominal hernia, Inflammatory bowel disease (IBD), Vascular intestinal disorders, Gallbladder and biliary disease, Pancreatitis, and other digestive diseases. According to the 11th revision of the International Classification of Diseases (ICD-11), we have analyzed six major digestive diseases in China: Cirrhosis and other chronic liver diseases (DA26.0, DB90-99, ME10), Upper digestive system diseases (DA60, DA63, DA61, DA22), Appendicitis (DB10-11.6, DB1Y-Z), Paralytic ileus and intestinal obstruction (DC30-35, DC3Y-Z), Gallbladder and biliary disease (DA91, DB30), and Inflammatory bowel disease (DD70-72, DD7Y-Z).

### 2.2 Data sources

This study utilizes data spanning from 2000 to 2019 sourced from the Global Burden of Disease (GBD) for China, the Chinese Health Statistical Yearbook covering the years 2000 to 2022, and a systematic analysis of epidemiological data on digestive diseases in China.

### 2.3 Statistical analysis

The assessment of the burden associated with digestive diseases involved calculating Age-Standardized Rates for incidence (ASIR), death (ASDR), Years of Life Lost (ASYLLs), Years Lived with Disability (ASYLDs), and Disability-Adjusted Life Years (ASDALYs). To illustrate the temporal trend in the burden of digestive diseases from 2000 to 2019, we calculated the Estimated Annual Percentage Changes (EAPC). The 95% Uncertainty Interval (95% UI) used in the estimation process indicates the level of data sparsity.

Age-Period-Cohort (APC) models are used to analyze the impact of age, period, and cohort on diseases. APC models initially gained significant traction in the field of cancer surveillance (9, 10), primarily being employed for conducting comparative risk analyses (11, 12). Presently, it has gained widespread application in examining population trends related to disease incidence and death rates (13–17), as well as in forecasting future disease burdens (18, 19). This model has evolved into a prominent statistical tool, extensively employed to uncover hidden information within death rates. It provides insights into the historical risk of death within a population and assesses the accumulation of health risks since birth (20). During the APC analysis, we categorized disease data into 18 age groups with 5-year intervals, based on age. Simultaneously, the population data was divided into four cycle groups at 5-year intervals spanning from 2000 to 2020. Our analysis was conducted using the Age-Period-Cohort Web Tool (21).

## 3 Results

### 3.1 Incidence trends of digestive diseases in Chinese residents

In comparison to the year 2000, there was a significant increase in the number of reported cases of digestive disease among Chinese residents in 2019. From 2000 to 2020, the number of incident cases of Gallbladder and biliary diseases experienced the greatest increase, rising from 9,902,000 cases to 18,642,000 cases, reflecting a 88.26% increase. And the incidence of Inflammatory bowel disease (IBD) increased from 29,300 to 51,400, marking a remarkable growth of approximately 75.43%. Additionally, the number of incident cases of Cirrhosis and other chronic liver diseases showed a small increase, from 345,000 cases to 409,000 cases, representing a 18.55% rise (Table 1).

**Table 1:** Incidence of digestive disease in China from 2000 to 2019.

	2000		2019		
Digestive diseases	Incident cases, in millions (95% UI)	ASR per 100,000 NO. (95% UI)	Incident cases, in millions (95% UI)	ASR per 100,000 NO. (95% UI)	EAPC NO. (95% UI£^©^)
Overall	47.811£¨(43.935–52.055)	3623.4 (3341.8–3,922)	68.153 (62.604–74.312)	3786.1 (3492.5–4,118)	0.22 (0.11–0.32)
Disorders					
Cirrhosis and other chronic liver diseases	0.345 (0.255–0.441)	23.6 (17.6–30.3)	0.409 (0.308–0.514)	22.5 (17.6–27.6)	−0.31 (−0.4−0.21)
Upper digestive system diseases	32.681 (28.968–36.59)	2445.4 (2186.6–2729.7)	42.328 (37.492–47.79)	2,343 (2082.7–2635.6)	−0.41 (−0.68−0.14)
Gallbladder and biliary diseases	9.902 (8.327–11.657)	764.1 (651.5–895)	18.642 (15.531–22.54)	983.2 (828–1166.4)	1.74 (1.4–2.08)
Appendicitis	2.061 (1.578–2.671)	148.8 (114.2–191.2)	2.486 (1.971–3.055)	177 (139–221)	0.99 (0.95–1.04)
Paralytic ileus and intestinal obstruction	1.182 (1.133–1.232)	100.2 (96.4–103.9)	1.683 (1.621–1.747)	99.8 (96.6–103.1)	−0.14 (−0.28–0)
Inflammatory bowel disease	0.029 (0.025–0.034)	2.02 (1.8–2.4)	0.051 (0.043–0.061)	3.01 (2.6–3.5)	2.06 (2–2.12)

Between 2000 and 2019, the age-standardized incidence rate (ASIR) of digestive diseases in China increase from 3623.4 to 3786.1 per 100,000, with an Estimated Annual Percentage Change of 0.22 (95% UI 0.11 to 0.32). The ASIR of Inflammatory bowel disease rose from 2.02 per 100,000 to 3.01 per 100,000, EAPC of 2.06 (2 to 2.12). The ASIR of Gallbladder and biliary diseases increased from 764.1 per 100,000 to 983.2 per 100,000, representing an EAPC of 1.74 (1.4 to 2.08). Appendicitis’s ASIR increased from 148.8 per 100,000 to 177 per 100,000, showing an estimated annual percent change (EAPC) of 0.99 (0.95 to 1.04) (Table 1).

However, the average annual incidence rates of Paralytic ileus and intestinal obstruction, Cirrhosis and other chronic liver diseases, as well as Upper digestive system diseases, all exhibited a declining trend. The Age-Standardized Incidence Rate of upper digestive system diseases experienced a significant decline, decreasing from 2445.4 to 2,343 per 100,000, with an EAPC of −0.41 (−0.68 to −0.14) (Table 1).

### 3.2 DALY, YLL, and YLD of digestive diseases among Chinese residents (2000–2019)

Between 2000 and 2019, the estimated annual percentage change of YLLs and DALYs for digestive diseases both demonstrated a decreasing trend. The standardized DALY rate for digestive diseases decreased significantly, dropping from 974.4 per 100,000 to 538.73 per 100,000, with EAPC of −3.38% (95% UI -3.54 to −3.21). Notably, the EAPC for Paralytic ileus and intestinal was −5.1% (−5.25 to −4.94), with the largest decrease, from 80.1 per 100,000 to 31.62 per 100,000. The EAPC for gallbladder and biliary tract diseases showed the smallest decrease, at −1.5% (−1.66 to −1.34), declining from 118.9 per 100,000 to 87.9 per 100,000 (Table 2).

**Table 2:** DALY of digestive disease in China from 2000 to 2019.

	2000		2019		
Digestive diseases	DALYs, in millions (95% UI)	Age-standardized DALY rate per 100,000 (95% UI)	DALYs, in millions (95% UI)	Age-standardized DALY rate per 100,000 (95% UI)	EAPC NO. (95% UI£^©^)
overall	11.699 (10.59–12.92)	974.4 (889.1–1069.6)	10.017 (8.549–11.49)	538.73 (463.3–615.64)	−3.38 (−3.54−3.21)
Disorders					
Cirrhosis and other chronic liver diseases	5.516 (5.041–6.045)	428.5 (391–468.3)	4.343 (3.641–5.146)	217.8 (183.4–256.9)	−3.99 (−4.22−3.77)
Upper digestive system diseases	2.518 (2.092–3.091)	211.7 (177.3–255.8)	2.324 (1.845–3.031)	124.5 (99–162.2)	−3.09 (−3.33−2.86)
Gallbladder and biliary diseases	1.438 (1.038–1.962)	118.9 (88.1–158.2)	1.671 (1.153–2.361)	87.9 (61.1–122.9)	−1.5 (−1.66-−1.34)
Appendicitis	0.127 (0.109–0.151)	10.6 (9.2–12.5)	0.065 (0.052–0.081)	4.31 (3.42–5.34)	−4.86 (−5.28−4.44)
Paralytic ileus and intestinal obstruction	0.725 (0.654–0.821)	80.1 (72.2–90.2)	0.391 (0.333–0.459)	31.62 (26.83–36.36)	−5.1 (−5.25−4.94)
Inflammatory bowel disease	0.235 (0.179–0.276)	21.6 (16.2–25.1)	0.232 (0.179–0.291)	13.09 (10.29–16.31)	−2.94 (−3.14−2.74)

Age-standardized YLL rates for digestive diseases showed a significant decline from 774.2 per 100,000 to 360.01 per 100,000, with EAPC of −4.34% (95% UI −4.52 to −4.16). Remarkably, the EAPC in YLLs for digestive diseases exhibited varying degrees of reduction, with the most significant decrease observed in appendicitis at 7.61% (−8.03 to −7.19), decreasing from 8.8 per 100,000 to 2.14 per 100,000 (Table 3).

**Table 3:** Age-standardized YLL rates of digestive disease from 2000 to 2019 in China.

	2000	2019	
Digestive diseases	Age-standardized YLL rate per 100,000 NO. (95% UI)	Age-standardized YLL rate per 100,000 NO. (95% UI)	EAPC NO. (95% UI£^©^)
overall	774.2 (720.7–841.1)	3s60.01 (307.95–419.41)	−4.34 (−4.52−4.16)
Disorders			
Cirrhosis and other chronic liver diseases	420.4 (384–460.9)	211.5 (177.1–251.2)	−4.05 (−4.28−3.83)
Upper digestive system diseases	137.2 (124.2–164.7)	60.5 (51.4–70.9)	−4.58 (−4.87−4.3)
Gallbladder and biliary diseases	36.7 (26.7–40.9)	15.1 (12.4–20.2)	−4.82 (−5.18−4.47)
Appendicitis	8.8 (7.8–10.6)	2.14 (1.72–2.63)	−7.61 (−8.03−7.19)
Paralytic ileus and intestinal obstruction	77.5 (69.6–87.8)	29.02 (24.39–33.81)	−5.37 (−5.54−5.21)
Inflammatory bowel disease	16.2 (11.4–18.8)	6.02 (4.78–6.95)	−5.66 (−5.92−5.4)

In 2000, the standardized YLD rate for digestive diseases stood at 200.4 per 100,000, decreased to 178.72 per 100,000 by 2019, the EAPC of −0.62% (95% UI -0.74 to −0.51). The EAPC of YLDs for appendicitis and inflammatory bowel disease exhibited an increasing trend, whereas the ASYLDs for the remaining diseases continued to show a decreasing trend. IBD was increased from 5.4 per 100,000 to 7.07 per 100,000, the EAPC of 1.42% (1.35 to 1.48). Appendicitis experienced an increase, climbing from 1.8 per 100,000 to 2.16 per 100,000, the EAPC of 0.99% (0.94 to 1.04) (Table 4).

**Table 4:** Age-standardized YLD rates of digestive disease from 2000 to 2019 in China.

	2000	2019	
Digestive diseases	Age-standardized YLD rate per 100,000 NO. (95% UI)	Age-standardized YLD rate per 100,000 NO. (95% UI)	EAPC NO. (95% UI£^©^)
overall	200.4 (138.9–276.4)	178.72 (125.49–246.32)	−0.62 (−0.74−0.51)
Disorders			
Cirrhosis and other chronic liver diseases	8.1 (5.6–11.8)	6.3 (4.3–9.1)	−1.54 (−1.83−1.26)
Upper digestive system diseases	74.5 (47.1–112.4)	64.1 (39.7–100.7)	−0.99 (−1.26−0.71)
Gallbladder and biliary diseases	82.1 (52.2–121.7)	72.9 (46.3–107.5)	−0.49 (−0.63−0.34)
Appendicitis	1.8 (1.2–2.6)	2.16 (1.38–3.09)	0.99 (0.94–1.04)
Paralytic ileus and intestinal obstruction	2.6 (1.7–3.5)	2.59 (1.75–3.54)	−0.11 (−0.23–0.01)
Inflammatory bowel disease	5.4 (3.6–7.6)	7.07 (4.64–9.86)	1.42 (1.35–1.48)

### 3.3 Incidence and DALY rates stratified by age and sex in 2019

**Figure 1:**
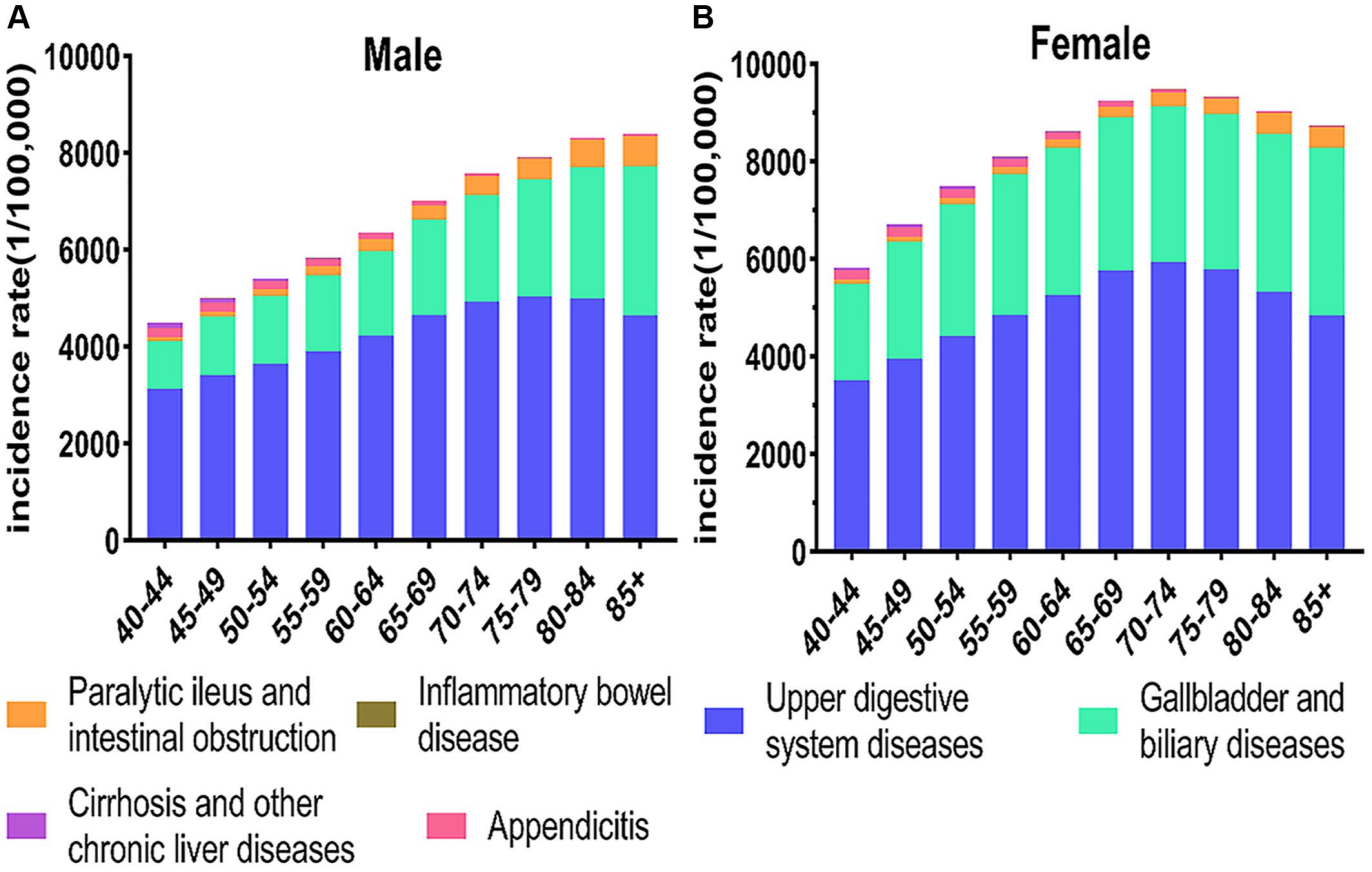
Incidence of digestive disease by age and gender in China, 2019. **(A)** The incidence of male. **(B)** The incidence of female.

Among populations aged 40 and above, gender-specific variations in incidence become apparent, revealing a higher incidence rates of digestive diseases in women compared to men. In males, the incidence of digestive diseases progressively increases with advancing age. Among females, the incidence of digestive diseases reaches its peak in the 70–74 age group before gradually declining with age. Upper digestive system diseases have the highest incidence rates, the peaking in the 70–79 age group before gradually declining (Figure 1).

**Figure 2:**
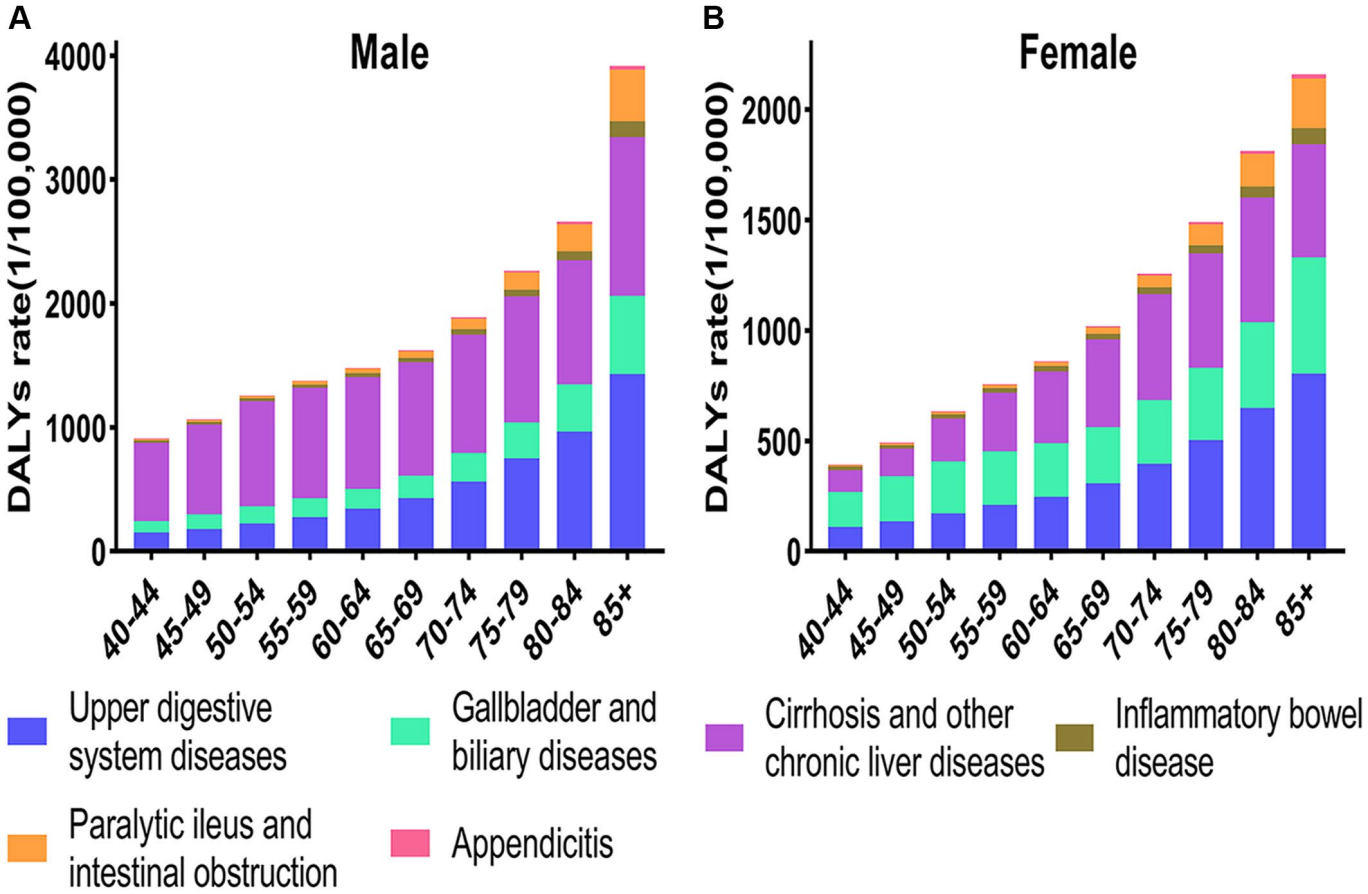
Age-standardized DALY rates of digestive diseases in China by age and gender, 2019. **(A)** The DALYs of male. **(B)** The DALYs of female.

Concerning the DALY rates for digestive diseases among populations aged 40 and older, it exhibits increase with advancing age. From a gender perspective, DALY rates for digestive diseases consistently demonstrated higher values in men than in women across all age groups. Upper digestive system diseases and cirrhosis collectively constituted for a higher percentage of DALY rates among digestive diseases (Figure 2).

### 3.4 Impact of age, period, and cohort on death rate from digestive diseases

**Figure 3:**
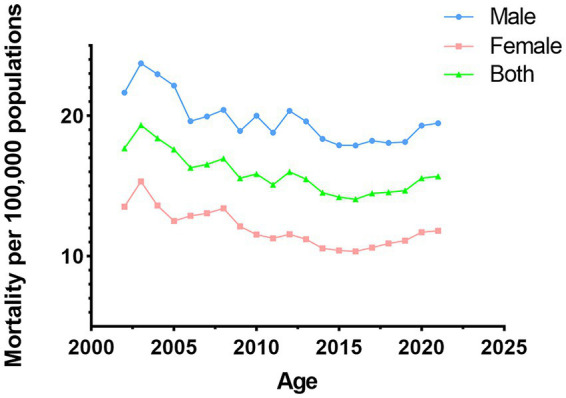
Gender differences in death rates from digestive diseases in China.

In China, the death rates for digestive diseases has consistently decline over time. Analyzing the data through a gender lens reveals that the death rates for digestive diseases has consistently been higher in men than in women. The males death rates reached its lowest point in 2018, after which it began to increase. The death rates among females reached their lowest point in 2016, followed by a gradual increase (Figure 3).

**Figure 4:**
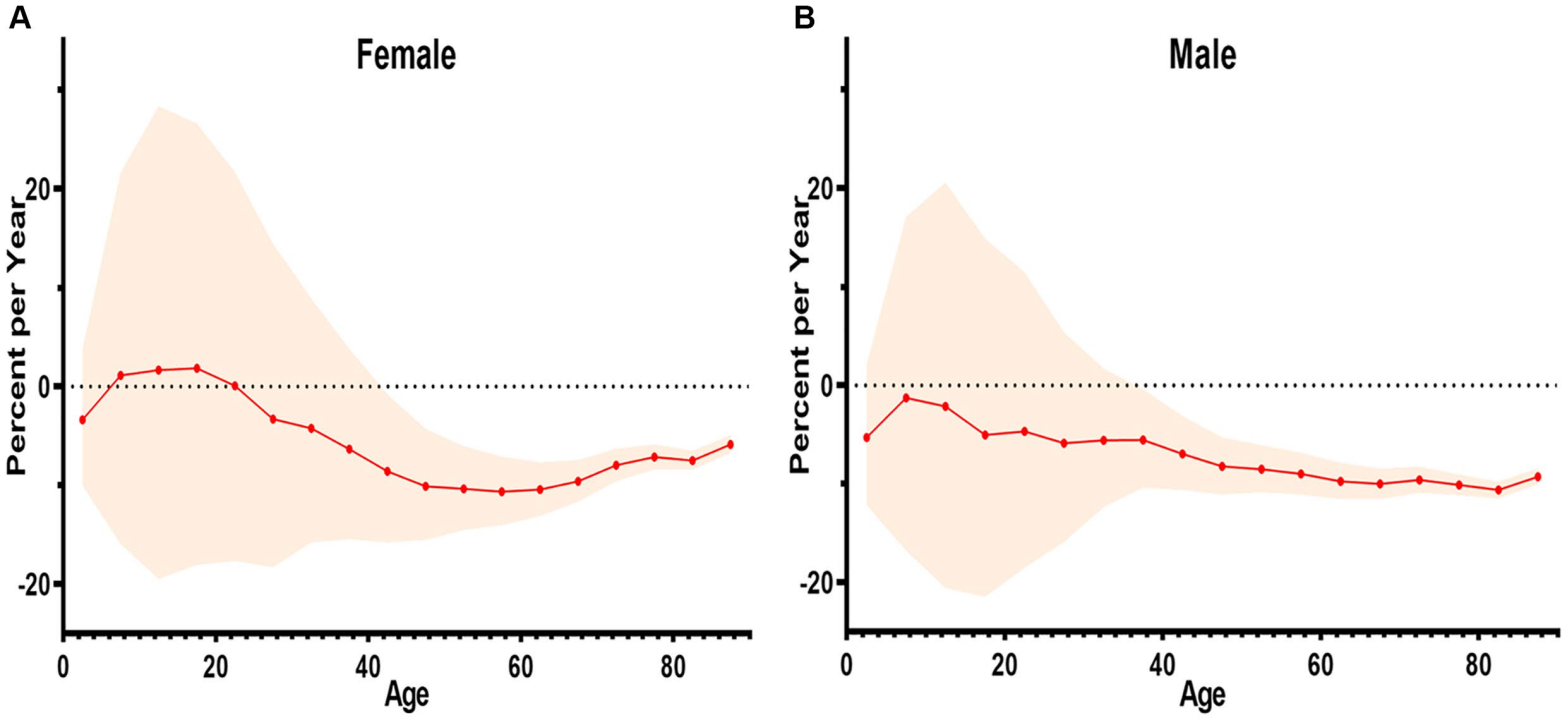
The net and localized drift of mortality from digestive diseases in China, 2002–2022. **(A)** The localized drift of female. **(B)** The localized drift of male.

In study, the net drift for males is −7.26%, and for females, it is −6.458%, indicating a statistically significant difference. The localized drift values observed for both males and females in China from 2002 to 2022 indicate a general decreasing trend in the death rates of digestive disease across all age groups. For females, the drift value exceeds zero only within the 5–20 age range, followed by a rapid decline reaching its lowest point around the age of 60. Subsequently, there is a gradual rise beyond the 60 year that never surpasses zero. In contrast to females, the males drift values consistently remain below zero across all age groups, indicating a persistent decline in the death rates digestive disease among males (Figure 4).

**Figure 5:**
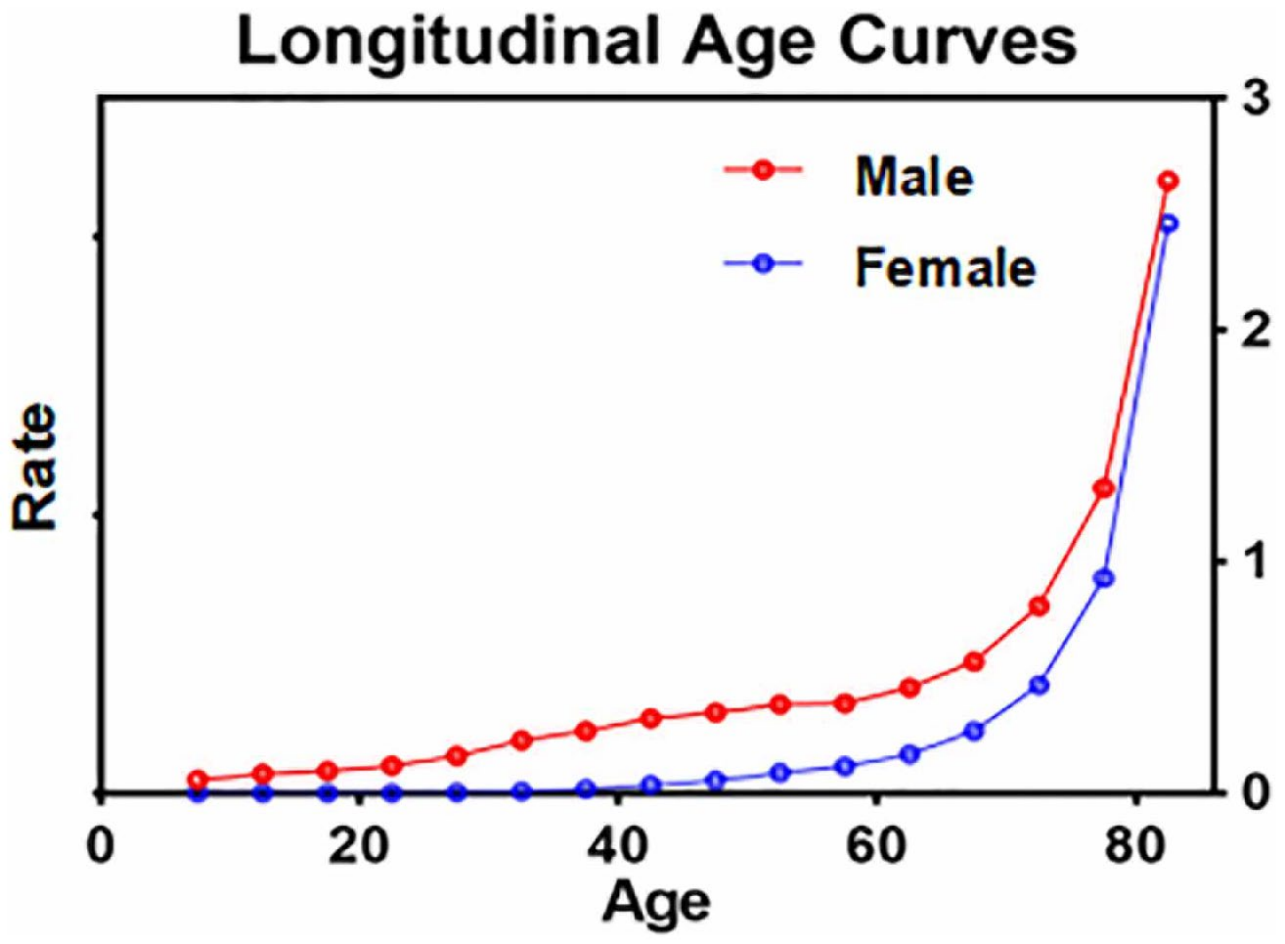
Age-related death rate trends in digestive diseases among Chinese residents, 2002–2022.

Age effects were illustrated using Longitudinal Age Curves in this study, emphasizing death rates related to digestive diseases across different age groups. In the same birth cohort, the death rates for digestive diseases gradually increased with age. Males experienced a significantly greater increase than females (Figure 5).

**Figure 6:**
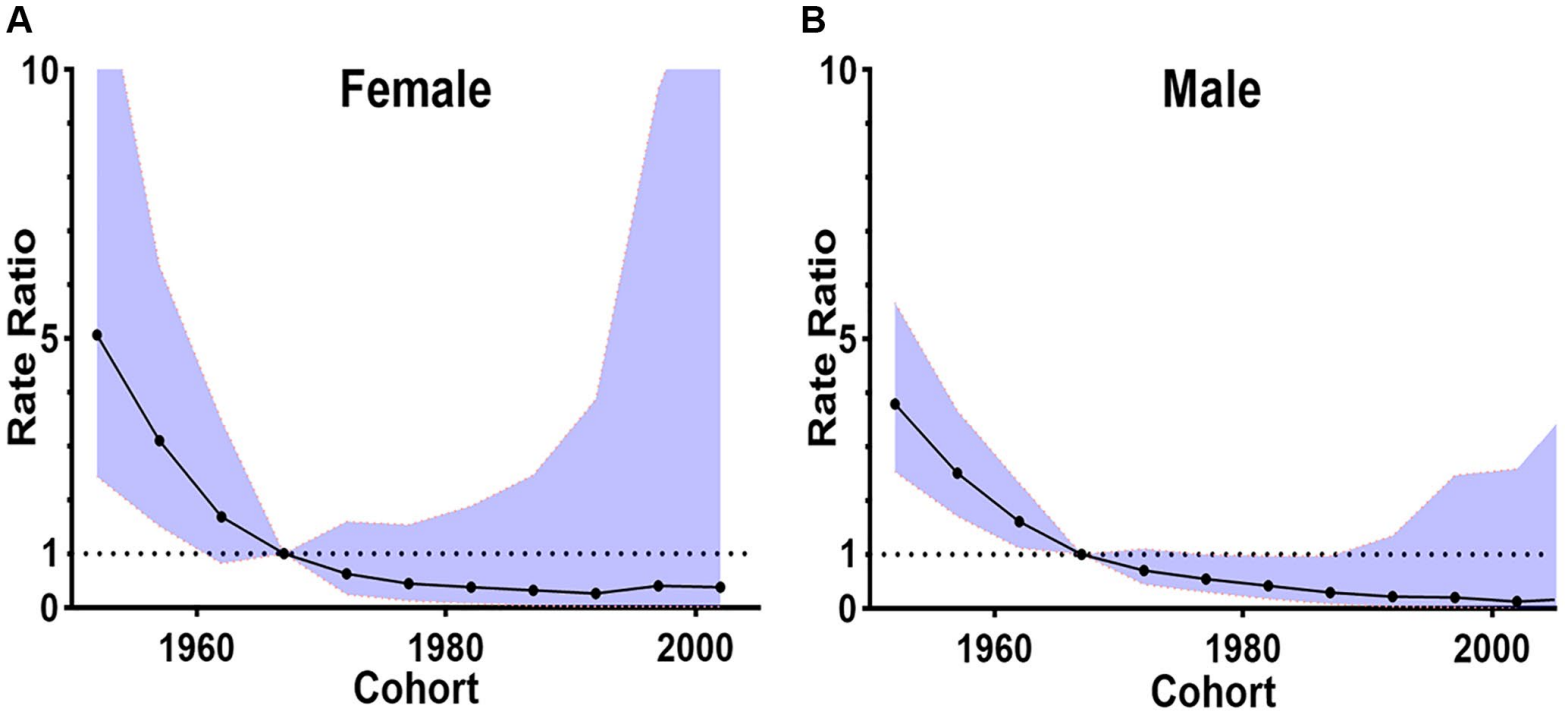
The birth cohort effects in China by gender, 2002–2020. **(A)** The cohort rate ratio of female. **(B)** The cohort rate ratio of male.

The birth cohort effect was demonstrated through the utilization of the Cohort Rate Ratio (RR). In this study, using 1965 as the reference point, the risk of death from digestive diseases was found to be higher among individuals aged 60 years and older, increasing with age. Conversely, the risk was lower for those under 60 years (Figure 6).

**Figure 7:**
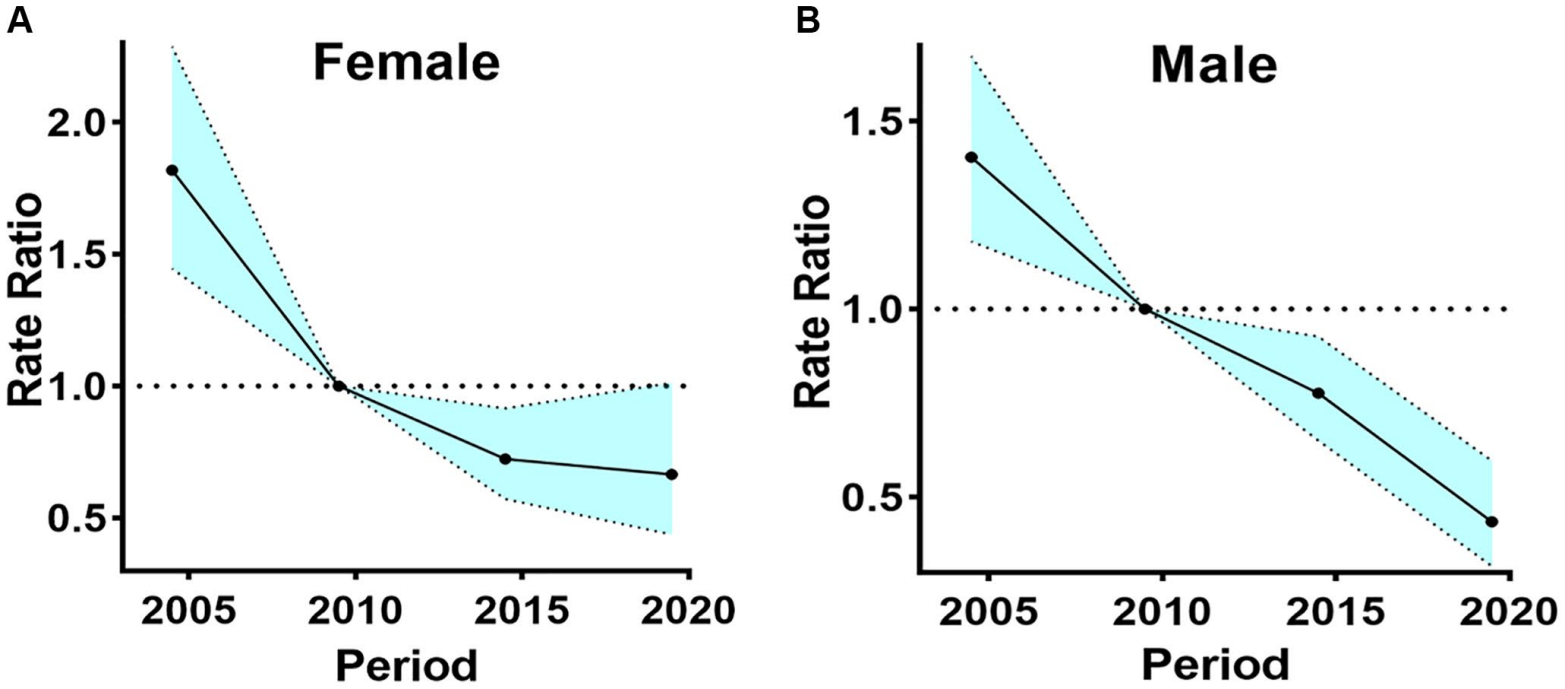
The period effects in China by gender, 2002–2020. **(A)** The period rate ratio of female. **(B)** The period rate ratio of male.

To study period effects, we use Period RR. RR > 1 indicates an increased relative risk of death compared to the reference period, while RR < 1 suggests a decreased relative risk of death relative to the reference period. In this study, the risk of death from digestive diseases has been decreasing for both males and females, with males experiencing a more significant decrease in risk. The risk of death for females exhibited a gradual decline, registering RR of less than 1 post-2010. Similarly, the risk of death for males has shown a steady decrease, with RR dipping below 1 after 2010 and reaching less than 0.5 by the year 2020 (Figure 7).

## 4 Discussion

The burden of digestive diseases in China corresponds is equivalent to the global burden of disease. Globally, there has been a sustained decline in the age-standardized incidence of cirrhosis and other chronic liver diseases, upper digestive system diseases, paralytic ileus, intestinal obstruction, and inflammatory bowel disease, as well as the DALY, YLL, and YLD, all exhibited a sustained decline (2). The age-standardized DALY rates and YLL rates associated with Appendicitis have witnessed a decline. However, there is a concurrent increase in the age-standardized incidence and YLD rates from 1990 to 2019 (22). The age-standardized incidence of gallbladder and biliary diseases has consistently increased (23).

Between 2000 and 2019, there was an upward trend in the age-standardized incidence rates of Inflammatory Bowel Disease, Gallbladder and Biliary Diseases of China. The global age-standardized incidence for Inflammatory Bowel Disease and Gallbladder and Biliary Diseases have consistently decreased (2). However, the age-standardized incidence of IBD in China is showing an upward trend. Cui et al. discovered that the rapid surge in incidence of inflammatory bowel disease (IBD) in China may be attributed to changes in environmental factors and the adoption of modern dietary patterns (24). Moreover, previous studies have demonstrated that Western dietary patterns, which are characterized by high levels of fat, fermentable carbohydrates, and protein from meat and fish, are associated with a higher incidence of IBD (25). Additionally, Park et al. found that an elevated body mass index is associated with an increased incidence of Gallbladder and biliary diseases (26).

In Asia, age-standardized incidence rates (ASIR) of IBD is on the rise in most countries. Japan had the highest ASIR than the country, while Thailand (0.12 per 100,000) had the lowest ASIR in 2019. Japan experienced a significant increase in ASIR, from 11.22 per 100,000 population to 19.65 per 100,000 population from 1990 to 2019 (27). The age-standardized incidence of IBD, encompassing both Crohn’s disease (CD) and ulcerative colitis (UC), experienced a significant decrease in Poland from 2009 to 2020. In Poland, the age-standardized incidence of CD decreased from 12.3 to 2.1, representing an 82.93% reduction. The age-standardized incidence of UC experienced an 86.21% decline, decreasing from 43.5 to 6 (28). The newly industrialized countries of Asia and Latin America have entered the second epidemiological stage (Acceleration in Incidence) in the global evolution of IBD in the 21st century. This stage is characterized by a rapid increase in the incidence of IBD. And the Western nations (North America and Europe) are in the third epidemiological stage (Compounding Prevalence): with a stabilizing or declining incidence of IBD (29). Reducing the disease burden of IBD in China necessitates healthcare systems to enhance their infrastructure and personnel readiness, ensuring an equitable, affordable, and accessible distribution of care for IBD patients (30).

The age-standardized death rate for digestive diseases exhibited a decline, decreasing from 46.67 per 100,000 in 1990 to 32.07 per 100,000 in 2019. The EAPC was −1.38 (95% UI −1.44 to −1.31). Besides Eastern Europe and Central Asia, other regions worldwide have also experienced a declining trend in the age-standardized death rates. Globally, the age-standardized death rates (ASDR) is significantly higher in men compared to women (2). The study demonstrates a correlation between the ASDR of digestive diseases in China and the global trend. In our findings, we observed males demonstrate higher death rates compared to females in China, which can be attributed to their engagement in high-risk occupations and behaviors, such as smoking and excessive alcohol consumption (6). These risk factors contribute to a heightened susceptibility to severe digestive diseases and increased death rates among males. With the Chinese government’s strict enforcement of anti-smoking and alcohol consumption policies, the death rates for males in China with digestive diseases has significantly decreased in recent years.

The study regarding age cohort period, death rates are significantly higher before the 5 year and after the 60 year compared to other age groups. It is speculated that these variations may be related to the physiological development, bodily functions, and dietary habits that are specific to each age group. Specifically, populations under 5 year of age may face unique health challenges due to immature organs and immune systems, which are exacerbated by environmental factors. Additionally, infantile hepatitis syndrome and children’s biliary atresia in children are potential factors that may contribute to these outcomes. In contrast, individuals over 60 year face a higher death rate, which could be influenced by age-related factors and dietary practices. In old age, individuals experience a general decline in organ function, including the digestive system. This decline results in diminished local immunity and defense capabilities within the digestive organs. Simultaneously, the functions of vital organ reserves weaken, exacerbated by environmental pollutants in the air, water, and food safety issues. Consequently, these factors contribute to an increased death rates in older adult individuals (31).

According to the latest 2020 census in China, the proportion of population aged 60 year and above has reached nearly 26%. China is currently undergoing a demographic transition characterized by an aging population. This demographic shift poses significant challenges for China, particularly in addressing the healthcare needs of its growing senior population, including conducting research on the incidence and trends of chronic diseases (32). To enhance protective measures against digestive diseases for both under 5-year-old and over 60-year-old age group, the Chinese government should intensify campaigns promoting the prevention and treatment of these diseases. The Chinese government should place emphasis on early prevention and treatment of digestive diseases, making concerted efforts to further reduce their incidence and mortality in China. Moreover, continuous education and policy support are essential for cultivate favorable dietary habits and promoting healthy lifestyles. For the older adult, it is crucial to promote regular daily exercise and encourage appropriate dietary habits and routines.

Nevertheless, it is imperative to acknowledge several limitations. Considering China’s extensive geographical diversity, dietary differences, economic disparities, and diverse healthcare conditions across provinces and ethnic groups, the impact on the incidence and mortality of digestive diseases can vary significantly. Subsequent research should explore the incidence and death rates of digestive diseases among diverse cities and provinces. Additionally, a comparative investigation of the risk factors associated with each cause of digestive disease is warranted.

## Data availability statement

The original contributions presented in the study are included in the article/supplementary material, further inquiries can be directed to the corresponding author.

## Author contributions

SG: Data curation, Formal analysis, Methodology, Writing – original draft, Writing – review & editing. YZ: Data curation, Formal analysis, Methodology, Writing – original draft. YW: Data curation, Methodology, Supervision, Visualization, Writing – original draft. XY: Data curation, Methodology, Supervision, Visualization, Writing – original draft. BC: Data curation, Formal analysis, Supervision, Visualization, Writing – review & editing. ZS: Data curation, Formal analysis, Supervision, Visualization, Writing – review & editing. XL: Conceptualization, Project administration, Software, Writing – review & editing.
